# Genome-Wide Identification and Functional Differentiation of Fatty Acid Desaturase Genes in *Olea europaea* L.

**DOI:** 10.3390/plants11111415

**Published:** 2022-05-26

**Authors:** Erli Niu, Song Gao, Wenjun Hu, Chengcheng Zhang, Daqun Liu, Guoxin Shen, Shenlong Zhu

**Affiliations:** 1 Zhejiang Academy of Agricultural Sciences, Hangzhou 310021, China; niuerli@zaas.ac.cn (E.N.); gaos@zaas.ac.cn (S.G.); guyuexingshi@163.com (W.H.); zccwsf@126.com (C.Z.); liudaqun@zaas.ac.cn (D.L.); guoxin.shen@ttu.edu (G.S.); 2 Key Laboratory of Digital Dry Land Crops of Zhejiang Province, Hangzhou 310021, China

**Keywords:** olive (*Olea europaea* L.), fatty acid desaturase, phylogenetic analysis, morphogenesis, stress response

## Abstract

Olive (*Olea europaea* L.) is a world-famous woody oil tree and popular for redundant unsaturated fatty acids. Fatty acid desaturase (FAD) genes are responsible for fatty acid desaturation and stress regulation but have not yet been identified in olive at the whole genome level. This study identified 40 and 27 FAD genes in the cultivated olive *O. europaea* cv. Farga and the wild olive *O. europaea* var. Sylvestris, respectively. Phylogenetic analysis showed that all the FAD genes could be classified into the soluble FAB2/SAD clade and membrane-bound clade, including ADS/FAD5, DES, FAD4, SLD, ω-6 and ω-3, with the high consistency of subcellular localization, motif composition and exon-intron organization in each group. FAD genes in olive showed the diverse functional differentiation in morphology of different tissues, fruit development and stress responses. Among them, *OeFAB2.8* and *OeFAD2.3* were up-regulated and *OeADS.1*, *OeFAD4.1* and *OeFAD8.2* were down-regulated under the wound, *Verticillium dahliae* and cold stresses. This study presents a comprehensive analysis of the FAD genes at the whole-genome level in olives and will provide guidance for the improvement of oil quality or stress tolerance of olive trees.

## 1. Introduction

Olive (*Olea europaea* L.) is an important economic tree and widely cultivated in more than 40 countries [1]. Olive oil is the only woody oil extracted from fresh olive fruits at low temperatures by mechanical method and contains a high amount of unsaturated fatty acids (UFAs) or oleic acids and various antioxidants, which has been the main part of Mediterranean diets since 6000 years ago [2,3]. Fatty acids are an important source of energy in daily food and are also important precursors for the formation of specific aromas during fruit development [4,5]. It is clear that, in addition to the main structural components of biological macromolecules, fatty acids also play a variety of physiological functions in plants. The physiological activity of fatty acids directly determines the fluidity of the cell membrane and can affect the resistance to temperature and water stress [6]. Besides, it would induce SA or ABA pathways as signal substances to participate in a variety of stress responses [7].

Fatty acids in plants can be divided into saturated fatty acids (SFAs) and UFAs according to the number of double bonds, and UFAs further contain monounsaturated fatty acids (MUFAs) and polyunsaturated fatty acids (PUFAs). According to the position of the double bonds, unsaturated fatty acids include ω-3, ω-6, ω-7 or ω-9 types [4]. The biosynthesis of fatty acids is a very complex process and involved in initial synthesis and desaturation [8,9]. In plastid, the precursor Acetyl-CoA converts into 16:0-CoA, 18:0-CoA and 18:1-CoA catalyzed by a series of synthase enzymes [8,10]. Subsequently, UFAs are synthesized in endoplasmic reticulum through lysophosphatidylcholine acyltransferase and desaturation [11]. Among the genes related to fatty acid biosynthesis, fatty acid desaturase (FAD) is a significant enzyme that can desaturate SFAs into UFAs [6]. Until now, the whole genome of FAD genes had been studied and resulted in 25, 19, 41, 84, 57, 45, 46, 30 and 33 FAD genes in *A. thaliana*, *O. sativa*, *G. max*, *B. napus*, *B. juncea*, *B. rapa*, *B. nigra*, *J. regia* and *M. sativa*, respectively [12,13,14,15,16,17]. All the FAD genes are typed as soluble or membrane-bound. The soluble FAD includes the only stearoyl-ACP desaturase (FAB2/SAD) group and specifically introduces a double bond at the Δ-9 position and desaturates 18:0-ACP into 18:1-ACP [18], while the membrane-bound FAD is involved in six groups: Acyl-CoA desaturase-like (ADS/FAD5), Sphingolipid Δ4 desaturase (DES), FAD4, Sphingolipid Δ8 desaturase (SLD), ω-3 and ω-6. ADS/FAD5 can introduce a double bond at the Δ-7 or Δ-9 position of the saturated acyl chain [19]. DES and SLD groups harbor the sphingolipid Δ-4 and Δ-8 FADs, respectively. FAD 4 group includes the Δ-3 FADs and catalyzes C16:0 to C16:1 [14,15]. ω-3 group introduces Δ-15 FADs and contain FAD3/FAD7/FAD8 genes, while ω-6 group introduces Δ-12 and contains FAD2/FAD6 genes [12,13,14,15,16,17].

Diversified FAD genes have been identified and confirmed in plants. FAB2/SAD is first key enzyme to increase the content of UFAs in plants [20]. RNA interference of the *GhSAD-1* gene in cotton greatly elevated the accumulation of C18:0 content from 2.3% to 39.8%, and silencing of the *GhFAD2-1* resulted in an increase in C18:1 content from 13.2% to 78.2% with the reduction in C18:2 from 58.5% to 3.7% [21]. CRISPR/Cas9-mediated genome editing of the *FAD2* gene in oilseed rape increased the contents of oleic acid compared with that in wild-type seeds [22]. As fatty acids are the main components of cell membranes, FAD genes also play important roles in adversity stresses, especially in response to low temperatures. Both *FAD2-3* and *FAD2-4* in cotton were induced by cold stress, but *FAD2-2* was not [23]. Over-expression of *FAD3* and *FAD7* in tomato exhibited a major increase in the desaturation ratio of C18:3/C18:2 to 18.21 and 5.02 in leaves, respectively, compared with the wild-type plants of 3.43 and also enhanced the resistance to cold stress [24]. *PtFAD2* transgenic *Populus tomentosa* resulted in the increase in C18:3 and C18:2 contents and the survival rate after freezing treatment [25]. In addition, the FAD genes have also been found to can affect the tolerance of salt, drought and osmotic stresses in plants [26,27].

As a major woody oil plant, it is essential to conduct the study of the FAD genes in olive. Contreras et al. [28] identified three ω-6 and three FAB2/SAD genes in olive and found that all the FAD genes displayed the constitutive expression patterns in different tissues and developing fruits. Transcriptome analysis of olive fruits obtained 31 and 12 FAD genes involved in the fatty acid metabolism [29,30]. Haplotype diversity analysis of 17 FAD genes showed three highly polymorphic SNPs and four haplotypes harboring differential oleic/linoleic acid ratios in olive [31]. The publication of olive genomic data [32,33,34] greatly benefits the identification of the FAD genes at the whole genome level. In this study, the novel FAD genes will be surveyed in the cultivated olive *O. europaea* cv. Farga and the wild olive *O. europaea* var. Sylvestris. Additionally, the various functions of FAD genes in morphology and stress response are analyzed with the available transcription data of olive. All this would provide gene resources for FAD gene utilization and benefit the improvement of olive traits through genetic manipulation.

## 2. Results

### 2.1. Genome-Wide Identification of FAD Genes in Olive

To obtain the FAD genes in olive at the genome-wide level, comprehensive analysis of the FAD genes in *A. thaliana*, *G. max* and *B. napus* [12,13,14] was conducted. It showed that all the FAD genes in different plants shared the notable domains PF00487, PF03405 or PF10520 in Pfam database [35]. Subsequently, the genome data of the cultivated olive *O. europaea* cv. Farga and the wild olive *O. europaea* var. Sylvestris were employed to search for the corresponding FAD genes in olive [32,33]. Taking the three domains as queries, the candidate genes were obtained in *O. europaea* cv. Farga and *O. europaea* var. Sylvestris using the HMMER tool [36]. After further verification by the online Motif Scan and SMART, 40 and 27 FAD genes were identified in *O. europaea* cv. Farga and *O. europaea* var. Sylvestris, respectively (Table 1), which displayed a large difference between the cultivated olive and wild olive.

### 2.2. Phylogenetic Analysis of FAD Genes in Different Species

**Figure 1:**
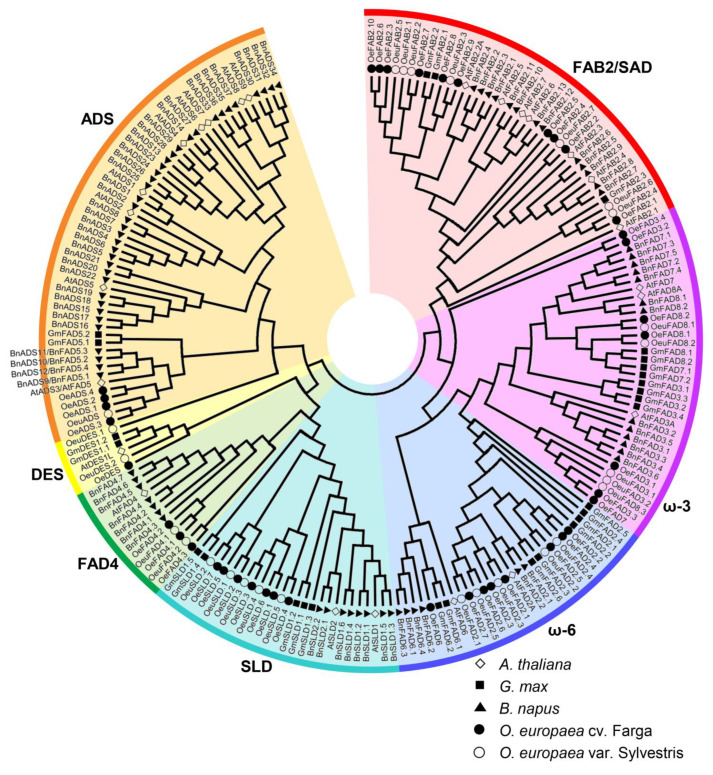
Phytogenetic tree of FAD genes in *A. thaliana*, *G. max*, *B. napus*, *O. europaea* cv. Farga and *O. europaea* var. Sylvestris. FAD genes in different species were shown by different icon shape. The gene IDs of FAD sequences in *A. thaliana*, *G. max* and *B. napus* referred from The Arabidopsis Information Resource [12], Chi et al. [13] and Xue et al. [14], respectively. The different groups were represented by differently colored bands.

To clarify the evolutionary relationships of the FAD genes, the overall protein sequences in diploid *A. thaliana*, *G. max*, *B. napus*, *O. europaea* cv. Farga and *O. europaea* var. Sylvestris were obtained to construct a phylogenetic tree with the maximum likelihood method [37]. According to the analysis results, the FAD genes were named in descending order of the gene ID (Table 1). As shown in Figure 1, all the FAD genes could be classified into two clades and seven groups. One clade included the soluble FAB2/SAD genes and shared the conserved domain PF03405. The other clade included the membrane-bound FADs, including six groups: ADS, DES, FAD4, SLD, ω-3 and ω-6. Of them, FAD4 had the domain PF10520 and the other FAD genes had the identical domain PF00487. *O. europaea* cv. Farga had 10 FAB2/SAD genes and 4 ADS, 1 DES, 3 FAD4, 7 SLD, 7 ω-3, 8 ω-6genes, while *O. europaea* var. Sylvestris had 7 FAB2/SAD genes and 1 ADS, 2 DES, 2 FAD4, 5 SLD, 5 ω-3, 5 ω-6 genes (Figure 1; Table 2). Except for the membrane-bound DES, the other membrane-bound FAD and soluble FAB2/SAD genes in the wild olive were less than those in the cultivated olive. Additionally, *OeuDES.1* was classified into the same branch with ADS group because of the incomplete amino acid sequence.

FAD genes in different plants showed clear differences (Table 2). The non-oil plants *A. thaliana* and *O. sativa* had 25 and 19 FAD genes, which were significantly less than that in the oil plants, and this occurred mainly in the membrane-bound FAD genes. In addition, diploid *G. max* and *B. rapa*/*B. oleracea* had 17 ω-6 and 20/18 ADS genes, respectively, displaying the obvious advantages in total FADs and membrane-bound FADs than the woody oil plants walnut and olive tree. The cultivated olive *O. europaea* cv. Farga had more FAD numbers in different clades than *J. regia*, except for DES and ω-3 genes. Among all the diploid oil plants, the cultivated olive showed the most FAB2/SAD (10) and SLD (7) genes, while the wild olive *O. europaea* var. Sylvestris had less FAD genes than the cultivated olive.

### 2.3. Gene Characters and Protein Subcellular Localization

Except for *OeuFAD4.2* distributed in chr7, the other FAD genes in *O. europaea* cv. Farga and *O. europaea* var. Sylvestris were distributed in short scaffolds (Table 1). Seven FAD genes in *O. europaea* cv. Farga (*OeFAB2.4*, *OeFAB2.5*, *OeADS.4*, *OeFAD3.2*, *OeFAD3.3*, *OeFAD3.4*, *OeFAD7*) and four FAD genes in *O. europaea* var. Sylvestris (*OeuFAB2.3*, *OeuFAB2.6*, *OeuDES.1*, *OeuFAD8.3*) had the amino acids length < 200 amino acids (a.a.). In *O. europaea* cv. Farga, amino acid length of the other FAD genes ranged from 208 (*OeFAB2.7*) to 461 (*OeFAD8.1*) a.a. with the predicted molecular weight (Mw) of 23921.35 (*OeFAB2.7*)-52736.20 (*OeFAD8.1*) Da. The amino acid length of the rest FAD genes in *O. europaea* var. Sylvestris ranged from 208 (*OeuFAB2.2*) to 473 (*OeuFAD8.2*) a.a. with the predicted MW of 23865.24 (*OeuFAB2*.2)-54190.85 (*OeuFAD8*.2) Da. Besides, the theoretical isoelectric points (pI) ranged from 5.38 (*OeFAB2.9*) to 9.36 (*OeADS.1* and *OeADS.3*) in *O. europaea* cv. Farga and 5.35 (*OeuFAB2.1*) to 9.18 (*OeuFAD8.1*) in *O. europaea* var. Sylvestris, respectively.

**Figure 2:**
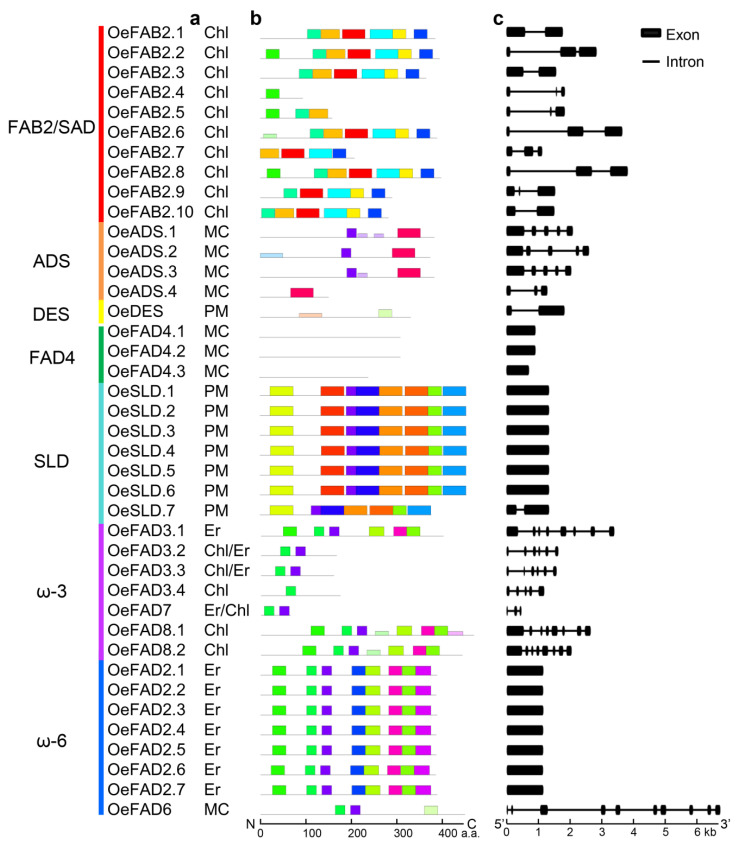
Analysis of subcellular localization, motif composition and exon/intron structure. Predicted subcellular localization (**a**), conserved motif (**b**) and gene structure (**c**) of FAD genes were conducted by Softberry (http://www.softberry.com/ accessed on 31 March 2022), MEME (https://meme-suite.org/meme/ accessed on 31 March 2022) and Gene Structure Display Server 2.0 (http://gsds.gao-lab.org/ accessed on 31 March 2022), respectively. The boxes with different colors represented various motifs. Chl, chloroplast; MC, membrane bound chloroplast; PM, plasma membrane; Er, endoplasmic reticulum.

To clarify the gene features of the FAD genes, subcellular localization, motif composition and exon/intron structure were also analyzed (Figure 2). All the FAB2 genes were located in chloroplast (Figure 2a). ADS and FAD4 genes were located in membrane bound chloroplast. DES and SLD were located in plasma membrane. ω-6 genes were located in endoplasmic reticulum or membrane-bound chloroplast and ω-3 genes were located in endoplasmic reticulum or chloroplast. The online MEME tool detected the top 20 conserved motifs in all FAD genes (Figure 2b). The genes in the same groups often presented similar conserved motifs, although there was no common motif among all FAD genes. Among the 20 conserved motifs, no motif was observed in FAD4 genes, indicating the uniqueness of FAD4 proteins. The analysis of the exon-intron structure further showed that FAB2/SAD, ADS, DES, ω-3 genes had 2–3, 3–5, 2 and 3–8 exons, respectively (Figure 2c). FAD4, SLD and ω-6 genes had only 1 exon, except for *OeSLD.7* and *OeFAD6* genes that contained 2 and 10 exons. The subcellular localization, motif composition and exon/intron structure displayed the high consistency among the clustered groups of FAD genes.

### 2.4. The Roles of FAD Genes in Olive Morphology

**Figure 3:**
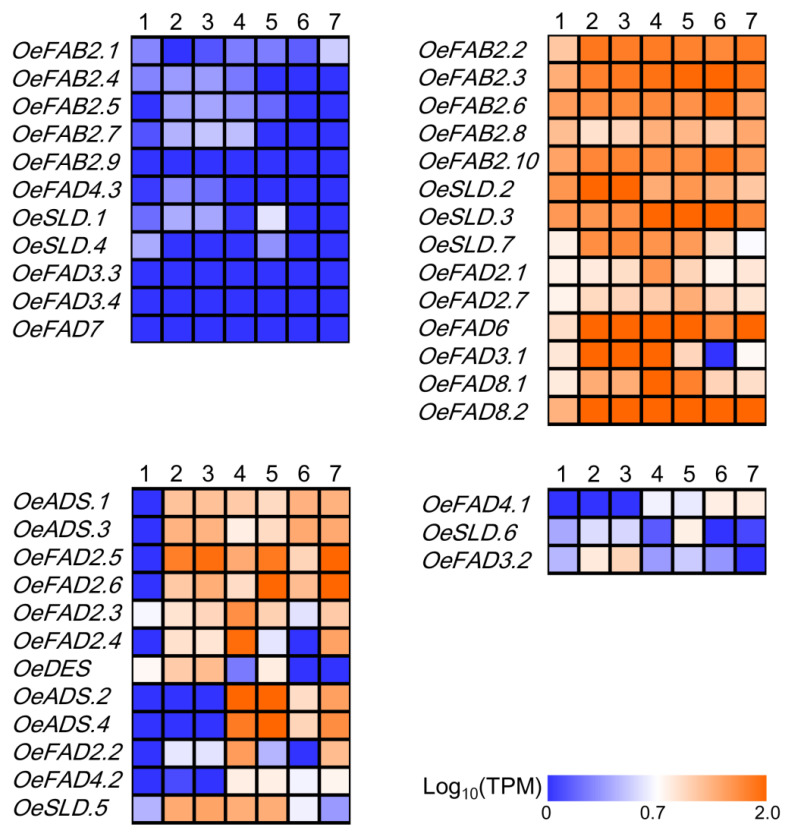
Different tissue expression profiles of FAD genes in olive. Transcriptome data of different tissues (PRJEB4992) were obtained from NCBI and included five tissues recorded as 1: roots, 2/3: leaves (young/old), 4: flowers, 5: flower buds, 6/7: fruits (green/immature). The log_10_(TPM) was calculated as the expression level. TPM, Transcripts Per Million. Four expression patterns of FAD genes in olive morphology were shown including the genes with TPM <5.0 in all five tissues, genes with TPM ≥5.0 in five tissues, genes with TPM ≥5.0 in two, three or four tissues and genes with TPM ≥5.0 in single tissue.

Based on the available transcriptome data of *O. europaea* cv. Farga involving in the morphology of olive (PRJEB4992), the expression patterns of FAD genes were detected in five tissues including roots, leaves (young/old), flowers, flower buds and fruits (green/immature) (Figure 3). The 44 FAD genes displayed four major types of expression patterns. A total of 11 genes (*OeFAB2.1*, *OeFAB2.4*, *OeFAB2.5*, *OeFAB2.7*, *OeFAB2.9*, *OeFAD4.3*, *OeSLD.1*, *OeSLD.4*, *OeFAD3.3*, *OeFAD3.4*, *OeFAD7*) had lower expression levels with the TPM < 5.0 in above five tissues. A total of 14 genes (*OeFAB2.2*, *OeFAB2.3*, *OeFAB2.6*, *OeFAB2.8*, *OeFAB2.10*, *OeSLD.2*, *OeSLD.3*, *OeSLD.7*, *OeFAD2.1*, *OeFAD2.7*, *OeFAD6*, *OeFAD3.1*, *OeFAD8.1*, *OeFAD8.2*) displayed constitutive expression patterns and were highly accumulated in five tissues with the TPM ≥5.0. A total of 12 genes (*OeADS.1*, *OeADS.3*, *OeFAD2.5*, *OeFAD2.6*, *OeFAD2.3*, *OeFAD2.4*, *OeDES*, *OeADS.2*, *OeADS.4*, *OeFAD2.2*, *OeFAD4.2*, *OeSLD.5*) had the higher expression levels (TPM ≥5.0) in two, three or four tissues. Three genes showed the predominant expression in single tissue. They were *OeFAD4.1*, *OeSLD.6* and *OeFAD3.2,* which had the transcription accumulation in fruits (green/immature), flower buds and leaves (young/old), respectively.

**Figure 4:**
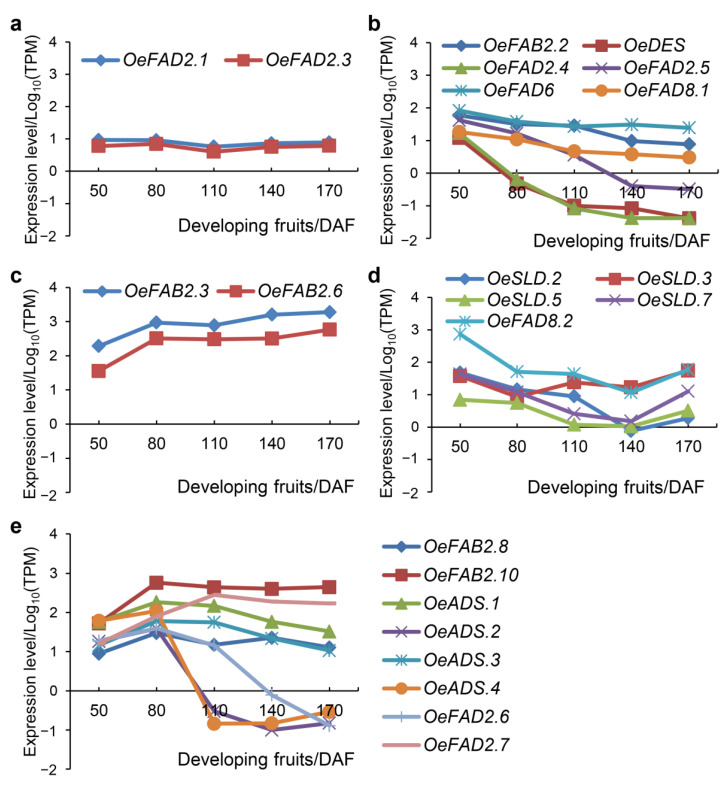
Fruits developing expression trends of FAD genes in olive. Transcriptome data of different tissues (PRJNA514943) were obtained from NCBI and included 50, 80, 110, 140, 170 DAF of olive fruits development stages. The log_10_(TPM) was calculated as the expression level. DAF, days after fertilization; TPM, Transcripts Per Million. Five expression patterns of FAD genes in developing olive fruits were shown including the genes had a relatively constant (**a**), continuous declining (**b**), continuous increasing (**c**) expression trend, and the genes with the expression levels decreased in the initial stage and then increased in the later stage (**d**) or increased in the initial stage and decreased in the later stage (**e**).

Different fruits’ development stages of *O. europaea* cv. Frantoio (PRJNA514943) were also obtained to elucidate the expression profiles of the FAD genes (Figure 4). Following 50, 80, 110, 140 and 170 days after fertilization (DAF) of the fruits’ development stages, 17 genes had an expression level of <5.0 in all detected fruits. Among the rest of the FAD genes, the expression levels of two genes (*OeFAD2.1* and *OeFAD2.3*) did not change significantly (Figure 4a). Six genes (*OeFAB2.2*, *OeDES*, *OeFAD2.4*, *OeFAD2.5*, *OeFAD6*, *OeFAD8.1*) and two genes (*OeFAB2.3*, *OeFAB2.6*) showed a continuous declining or increasing expression trend, respectively (Figure 4b,c). The expression levels of five genes (*OeSLD.2*, *OeSLD.3*, *OeSLD.5*, *OeSLD.7*, *OeFAD8.2*) decreased in the initial stage of fruit development and then increased at 140-170 DAF (Figure 4d). On the contrary, the expression levels of eight genes (*OeFAB2.8*, *OeFAB2.10*, *OeADS.1*, *OeADS.2*, *OeADS.3*, *OeADS.4*, *OeFAD2.6*, *OeFAD2.7*) increased in the initial stage of fruit development but decreased in the later stage (Figure 4e).

### 2.5. Stress Response of FAD Genes in Olive

**Figure 5:**
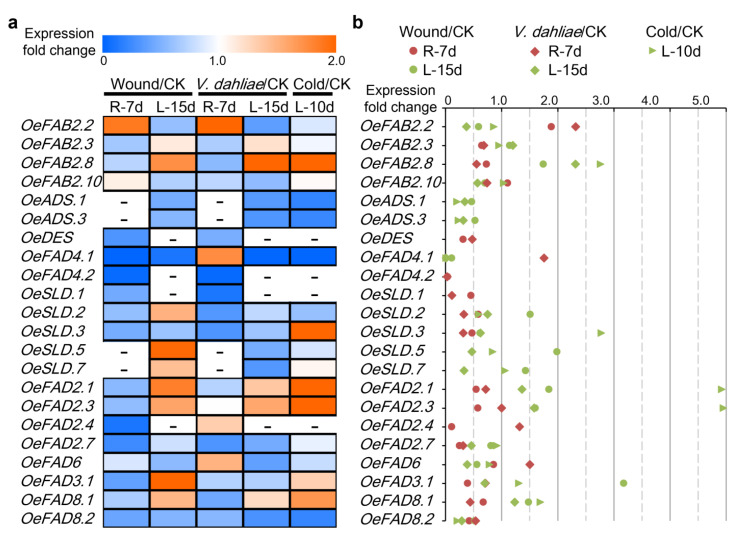
Expression heatmaps (**a**) and values (**b**) of FAD genes induced by different stresses in olive. Stress-induced expression profiles of FAD genes under wound, *Verticillium dahlia* and cold (PRJNA256033) were obtained from NCBI. Wound and *V. dahliae* were involved in the roots (R) and leaves (L) after 7 and 15 days of stress treatment, and cold was involved in the leaves after 10 days of stress treatment. The Transcripts Per Million (TPM) was calculated as the expression level and the up-regulated or down-regulated genes were identified with the fold change of stress/CK ≥1.5 or ≤0.5.

Previous studies showed that FAD genes played a positive role in adversity stress. The transcriptome data of stress treatments including wound, *Verticillium dahlia* and cold (PRJNA256033) were downloaded for further analysis (Figure 5a,b). In total, 18 genes showed the expression levels <5.0 in all detected samples. Two genes (*OeFAB2.8* and *OeFAD2.3*) were up-regulated under all the stress of wound (roots or leaves), *V. dahlia* (roots or leaves) and cold (leaves) with ≥1.5-fold change. *OeFAB2*.8 was highly expressed with 1.74-, 2.31- and 2.76-fold change in 15 d of leaves under wound stress, 15 d of leaves under *V. dahlia* stress and 10 d leaves under cold stress than in the respective controls. *OeFAD2.3* was highly expressed with 1.59-, 1.58- and 4.95-fold change in 15 d of leaves under wound stress, 15 d of leaves under *V. dahlia* stress and 10 d leaves under cold stress than in the respective controls. *OeFAB2.2* and *OeFAD2.1* were up-regulated under two stress treatments with ≥1.5-fold change. *OeFAB2*.2 was highly expressed with 1.88- and 2.31-fold change under wound and *V. dahlia* stress, while *OeFAD2.1* was highly expressed with 1.84- and 4.92-fold change under wound and cold stress. Six genes (*OeFAD4.1*, *OeSLD.3*, *OeSLD.5*, *OeFAD6*, *OeFAD3.1* and *OeFAD8.1*) were up-regulated under individual stress treatment with ≥1.5-fold change.

Down-regulated genes after stress treatment were further screened with the fold change ≤0.5 (Figure 5a,b). Three FAD genes (*OeADS.1*, *OeFAD4.1* and *OeFAD8.2*) were down-regulated under all the three stresses in roots or leaves. Five genes *OeDES*, *OeFAD4.2*, *OeSLD.1*, *OeSLD.3* and *OeFAD2.7* were down-regulated under wound and *V. dahlia* stress compared to the respective controls, while only *OeADS.3* was down-regulated under *V. dahlia* and wound stress. In addition, eight FAD genes *OeFAB2.2*, *OeSLD.2*, *OeSLD.5*, *OeSLD.7*, *OeFAD2.4*, *OeFAD6, OeFAD3.1* and *OeFAD8.1* were down-regulated under individual stress with ≤0.5-fold change. Overall, it seemed that wound, *V. dahlia* and cold stress had more FAD genes down-regulated than up-regulated.

## 3. Discussion

### 3.1. FAD Genes in Different Plants

FAD genes are essential factors which desaturate saturated fatty acids into unsaturated fatty acids for oil plants [6]. Until now, they have been identified and characterized in non-oil plants Arabidopsis (25) and rice (19) and oil plants soybean (41), rapeseed (84) and walnut (30) among others [12,13,14,15,16,17]. Olive is the only plant which extracts oil from its fresh fruits and is favored by consumers for redundant UFAs [38,39]. In this study, 40 and 27 FAD genes were retrieved in the cultivated olive *O. europaea* cv. Farga and the wild olive *O. europaea* var. Sylvestris, respectively. All the FAD genes could be distinguished as the soluble FAB2/SAD genes and membrane-bound FADs, including ADS, DES, FAD4, SLD, ω-3 and ω-6 [12,13,14,15,16,17] (Figure 1, Table 1 and Table 2). Protein analysis revealed that the former shared the domain PF03405 and the latter shared PF00487, except for FAD4, which shared PF10520. In addition, each group of the FAD genes was also highly conserved in terms of subcellular localization, motif composition and exon/intron structure, indicating the uniqueness within the group. Phylogenetic analysis showed that different plants had different groups of the FAD genes, which revealed that the FAD genes were descendants of an ancient duplication that occurred even before the separation of different plants.

Compared with the wild olive *O. europaea* var. Sylvestris, the cultivated olive *O. europaea* cv. Farga seems to have more FAD genes. This happens also for all FAD groups, but the DES group. The wild olive was considered to be an ancestor of the cultivated olive and related to Neolithic ancestors [40,41]. It could be inferred that the cultivated olive had undergone the expansion of the FAD genes and improved its trait of unsaturated fatty acids during the long-term evolution. Besides, wild olive retained more resistance to adverse environmental stress [33,41,42], so we could speculate that the unique FAD genes in wild olive may be associated with the resistance traits, but this needs further confirmation. As an example, the wild olive had two DES genes, while the cultivated olive just had one. The nucleic acid sequence of the FAD genes also had multiple genetic variations (synonymous and non-synonymous) between the wild and cultivated olives.

### 3.2. Functional Differentiation of FAD Genes in Olive

Previous studies showed that FAD genes in plants correspond to different morphogenesis, that is, some expressed specifically in a certain tissue or multiple organs. Especially, variation in locus or genes could directly affect the composition and proportion of fatty acids. In addition, diverse FAD genes also had a clear differentiation under different stresses [21,22,23,24,25,26,27]. At present, studies on the function of FAD in olive mainly focus on the transcription levels [28,29,30,31]. In this study, functional differentiation of FAD genes was studied at the genome-wide level from morphogenesis to stress response. The results showed that FAD genes in olive were involved in various metabolic processes of olive, including the development of different tissues and fruits, and the adversity induction of wound, *V. dahlia* and cold stresses (Figure 3, Figure 4 and Figure 5).

Soluble FAB2/SAD was the first enzyme in the determination of the ratio of saturated fatty acids to unsaturated fatty acids and could be enhanced by the stimuli of low temperature, fruit wounding, ethylene and CO_2_ [18,43]. On the whole, half of 10 FAB2/SAD genes in olive had lower expression levels and the rest displayed the constitutive expression patterns in different tissues with dynamic changes during the fruit’s development (Figure 3 and Figure 4). It seemed that the first step of desaturation in olive started up quickly in the 50–80 DAF and was proceeding during fruit development. Of them, *OeFAB2.2* was up-regulated in the roots under wound and *V. dahliae* stresses and down-regulated in the leaves under *V. dahlia*. *OeFAB2.8* was up-regulated in leaves under wound, *V. dahlia* and cold stresses (Figure 5). It insinuated the essential roles of FAB2/SAD in the stress defense of olive.

Among the membrane-bound FADs, four ADS genes and one DES gene exhibited higher expression levels (TPM > 5.0) in multiple tissues and were down-regulated by different stresses (Figure 3 and Figure 5). Besides, all four ADS genes had increasing expressions in the initial stage of fruit development, and decreasing in the later stage, while the DES gene had a continuous declining expression during the whole stage of fruit development (Figure 4). The FAD4 genes had lower expression levels in morphogenesis of olive and down-regulated by different stresses with the exception of *OeFAD4.1*, where it was up-regulated to 1.75-fold under *V. dahliae* treatment in leaves. Two SLD genes (*OeSLD.1* and *OeSLD.4*) had lower expression levels in different tissues and the rest of the SLD genes had higher expression in single or multiple tissues. During the stages of fruit development, the expression levels of *OeSLD.2*, *OeSLD.3*, *OeSLD.5* and *OeSLD.7* decreased in the initial stage and then increased in the later stage.

ω-3 and ω-6 genes have been verified and well-studied in different plants; they can catalyze C18:1 to C18:2 and C18:2 to C18:3, respectively, and included FAD3/FAD7/FAD8 and FAD2/FAD6 genes [12,13,14,15,16,17]. The mutants had a large increase in the corresponding UFAs [22,24,25], and the expression levels were induced by cold stress, salt, drought, osmotic and wound stresses [26,27,44]. In olive, the only FAD7 gene (*OeFAD7*) was rendered unexpressed in any case and the only FAD6 gene (*OeFAD6*) displayed a constitutive expression pattern with a slow descending expression during fruit development and was affected by *V. dahlia* and cold stresses (Figure 3, Figure 4 and Figure 5). Two FAD8 genes (*OeFAD8.1* and *OeFAD8.2*) expressed in multiple tissues but had different patterns in fruit development and stress response. Seven FAD2 and four FAD3 genes had diverse roles in tissues and fruits development or the stress induction (Figure 3, Figure 4 and Figure 5). Of them, *OeFAD2.1* and *OeFAD2.3* were highly stimulated by cold stress with the change fold of 4.92 and 4.95, respectively. FAD3 genes had lower expression during fruit development or under stress, insinuating their main functions in different tissues, except for *OeFAD3.1* which was down-regulated by 0.39-fold in roots and up-regulated by 3.17-fold in leaves under wound stress.

In conclusion, as the key enzyme that desaturates SFAs into UFAs, FAD genes also play important roles in the tolerance of different stresses. Here, we retrieved 40 and 27 FAD genes based on the genome sequences of the cultivated olive *O. europaea* cv. Farga and wild olive *O. europaea* var. Sylvestris, respectively. The FAD genes could be classified into the soluble FAB2/SAD genes with the conserved domain PF03405 and the membrane-bound FAD genes: ADS, DES, FAD4, SLD, ω-3 and ω-6 with the domain PF10520 or PF00487. The subcellular localization, motif composition and exon-intron organization had high consistency in each group. Phylogenetic analysis showed that FAD genes were descendants of an ancient duplication that occurred before the separation of different plants. The diploid oil plants had more FAD genes than non-oil plants, and *G. max* and *B. rapa*/*B. oleracea* displayed obvious advantages in total numbers of FAD and membrane-bound FAD genes than the woody oil plants walnut and olive tree. In particular, the cultivated olive had more FAD genes than the wild olive and the most FAB2/SAD and SLD genes among the diploid oil plants. All the FAD genes showed the diverse functional differentiation in morphology of different tissues, fruit development and the stress responses to wound, *V. dahlia* and cold. Finally, *OeFAB2.8* and *OeFAD2.3* were up-regulated under the three stresses with the fold change ≥1.5, while *OeADS.1*, *OeFAD4.1* and *OeFAD8.2* were down-regulated under the three stresses with the fold change ≤0.5. This study presents a comprehensive view of the FAD genes in olive at the whole-genome level and makes it possible to use FAD genes to improve the oil quality or stress tolerance of olive trees.

## 4. Materials and Methods

### 4.1. Database Search and FAD Genes Retrieval

The protein sequences of FAD genes in *A. thaliana*, *O. sativa*, *G. max*, *B. rapa*, *B. oleracea* and *J. regia* were obtained from The Arabidopsis Information Resource (TAIR: http://www.arabidopsis.org accessed on 31 March 2022), Chi et al. [13], Xue et al. [14] and Liu et al. [16], respectively. A unified analysis of the FAD genes of the three species identified three significant domains PF03405, PF00487 and PF10520 from Pfam database [35]. Taking the three domains as the queries, the corresponding proteins were retrieved from two olive genome data *O. europaea* cv. Farga [32] and *O. europaea* var. Sylvestris [33] using the HMMER software version 3.0 [36]. Subsequently, Motif Scan (http://myhits.isb-sib.ch/cgi-bin/PFSCAN accessed on 31 March 2022) and SMART (http://smart.embl-heidelberg.de/ accessed on 31 March 2022) were performed again to verify and detect all the conserved domains.

### 4.2. Phylogenetic Analysis

MEGA 7.0 software was employed to conduct the phylogenetic analysis with the Maximum Likelihood method based on the Jones-Taylor-Thornton (JTT) model [37]. Parameters were as follows. Test of phylogeny: bootstrap method, No. of bootstrap replications: 1000, substitutions model/method: JTT model, rates among sites: uniform rates, ML heuristic method: nearest-neighbor-interchange (NNI) and initial tree for ML: make initial tree automatically.

### 4.3. Physicochemical Character and Subcellular Localization Prediction

Theoretical isoelectric point (pI) and molecular weight (Mw) were calculated using the online website ExPasy (http://web.expasy.org/compute_pi/ accessed on 31 March 2022). The integral prediction of protein location was conducted by Softberry (http://www.softberry.com/ accessed on 31 March 2022). The gene feature visualization server Gene Structure Display Server 2.0 was used for gene exon/intron structure (http://gsds.gao-lab.org/ accessed on 31 March 2022). The conserved motif annotation was performed using the MEME program (https://meme-suite.org/meme/ accessed on 31 March 2022) with the following parameters: number of repetitions: any; maximum number of motifs: 10; the optimum motif widths: between 4 and 50 residues.

### 4.4. Expression Analysis of FAD Genes in Olive

For the expression analysis, we obtained the high throughput RNA-sequencing profiles of olive including the different tissues (PRJEB4992), fruits development (PRJNA514943) and stress-induced (PRJNA256033). With the reference genome *O. europaea* cv. Farga [32], the expression levels of genes were calculated as the Transcripts Per Million (TPM) by Cufflinks software with default parameters (http://cufflinks.cbcb.umd.edu/ accessed on 31 March 2022). Finally, the expression levels of FAD genes in different tissues and fruits development were recorded as log_10_(TPM) and the up-regulated or down-regulated genes under different stresses were identified with the fold change of stress/CK ≥ 1.5 or ≤ 0.5.

## Figures and Tables

**Table 1:** Gene IDs and characters of FAD genes in olive.

Cultivated/Wild Olive	Groups	Gene Name	Gene ID	Chr./Scaffold	Protein Length/a.a.	Molecular Weight/Da	Isoelectric Point
*O. europaea* cv. Farga	FAB2/SAD	*OeFAB2.1*	OE6A002165	s09619	386	43,985.23	6.2
		*OeFAB2.2*	OE6A012975	s05995	396	45,549.82	6.08
		*OeFAB2.3*	OE6A020845	s05960	366	41,893.6	5.44
		*OeFAB2.4*	OE6A024078	s03906	94	10,932.6	6.83
		*OeFAB2.5*	OE6A033129	s03906	159	18,196.6	5.96
		*OeFAB2.6*	OE6A048475	s02255	390	44,535.91	6.11
		*OeFAB2.7*	OE6A060676	s03906	208	23,921.35	8.45
		*OeFAB2.8*	OE6A089828	s09854	399	45,584.11	6.58
		*OeFAB2.9*	OE6A108617	s03845	291	33,386.07	5.38
		*OeFAB2.10*	OE6A118450	s09608	283	32,301.89	5.68
	ADS	*OeADS.1*	OE6A005264	s08189	384	43,921.63	9.36
		*OeADS.2*	OE6A080903	s00065	375	43,343.08	9.28
		*OeADS.3*	OE6A090227	s00065	384	43,987.69	9.36
		*OeADS.4*	OE6A120700	s08189	151	17,260.67	6.64
	DES	*OeDES*	OE6A026807	s03867	332	38,815.97	7.88
	FAD4	*OeFAD4.1*	OE6A064387	s09642	305	34,001.84	8.19
		*OeFAD4.2*	OE6A070143	s09790	305	33,954.76	8.19
		*OeFAD4.3*	OE6A082617	s07944	237	26,292.52	8.85
	SLD	*OeSLD.1*	OE6A013963	s00013	447	51,607.52	8.68
		*OeSLD.2*	OE6A023328	s07941	447	51,536.29	7.93
		*OeSLD.3*	OE6A037200	s07705	447	51,568.46	8.68
		*OeSLD.4*	OE6A037564	s00022	448	51,531.57	8.76
		*OeSLD.5*	OE6A087532	s09955	447	51,649.44	7.63
		*OeSLD.6*	OE6A107382	s05784	447	51,823.75	8.7
		*OeSLD.7*	OE6A114237	s01961	371	43,394.75	8.6
	ω-3	*OeFAD3.1*	OE6A024624	s02233	395	45,696.39	7.17
		*OeFAD3.2*	OE6A086562	s09646	164	18,813.28	7.77
		*OeFAD3.3*	OE6A109248	s06024	158	17,919.2	6.19
		*OeFAD3.4*	OE6A113752	s00046	172	19,727.44	6.7
		*OeFAD7*	OE6A117679	s04425	64	7269.06	6.08
		*OeFAD8.1*	OE6A074266	s07757	461	52,736.2	7.79
		*OeFAD8.2*	OE6A075849	s03965	436	49,806.14	9.18
	ω-6	*OeFAD2.1*	OE6A011870	s00121	383	43,916.5	8.73
		*OeFAD2.2*	OE6A019200	s05069	381	44,216.96	8.48
		*OeFAD2.3*	OE6A051290	s00121	383	43,907.49	8.73
		*OeFAD2.4*	OE6A067271	s07908	381	44,167.93	8.62
		*OeFAD2.5*	OE6A069627	s01964	381	44,182.9	8.46
		*OeFAD2.6*	OE6A085290	s05804	380	43,729.63	8.62
		*OeFAD2.7*	OE6A098403	s07710	383	44,064.09	9.01
		*OeFAD6*	OE6A116067	s07955	443	51,281.56	9.21
*O. europaea* var. Sylvestris	FAB2/SAD	*OeuFAB2.1*	Oeu009943	scaffold13793	366	41,906.65	5.53
		*OeuFAB2.2*	Oeu015714	scaffold1625	208	23,865.24	7.02
		*OeuFAB2.3*	Oeu024466	scaffold2105	187	21,435.4	6.53
		*OeuFAB2.4*	Oeu025716	scaffold2199	386	43,746.94	6.03
		*OeuFAB2.5*	Oeu040504	scaffold369	390	44,622.01	6.11
		*OeuFAB2.6*	Oeu048444	scaffold5181	106	12,242.2	8.88
		*OeuFAB2.7*	Oeu050331	scaffold564	407	46,693.26	6.03
	ADS	*OeuADS*	Oeu027810	scaffold2325	314	36,183.62	8.71
	DES	*OeuDES.1*	Oeu027812	scaffold2325	110	12,813.52	6.96
		*OeuDES.2*	Oeu053967	scaffold655	331	38,746.7	7.31
	FAD4	*OeuFAD4.1*	Oeu042929	scaffold41	305	34,047.89	8.24
		*OeuFAD4.2*	Oeu062594	chr7	237	26,199.36	8.62
	SLD	*OeuSLD.1*	Oeu003987	scaffold1142	447	51,536.29	7.93
		*OeuSLD.2*	Oeu009350	scaffold1349	447	51,753.64	8.33
		*OeuSLD.3*	Oeu036586	scaffold314	450	51,825.81	8.65
		*OeuSLD.4*	Oeu036587	scaffold314	447	51,588.45	8.68
		*OeuSLD.5*	Oeu063672	scaffold949	448	51,531.57	8.76
	ω-3	*OeuFAD3.1*	Oeu004670	scaffold1172	373	43,368.95	8.35
		*OeuFAD3.2*	Oeu015599	scaffold162	373	43,253.83	8.61
		*OeuFAD8.1*	Oeu004694	scaffold1173	436	49,840.16	9.18
		*OeuFAD8.2*	Oeu033588	scaffold281	473	54,190.85	8.14
		*OeuFAD8.3*	Oeu050958	scaffold58	63	7167.97	6.47
	ω-6	*OeuFAD2.1*	Oeu007766	scaffold1284	383	44,071.13	9.09
		*OeuFAD2.2*	Oeu013924	scaffold1547	381	44,182.9	8.46
		*OeuFAD2.3*	Oeu033739	scaffold283	383	43,948.5	8.73
		*OeuFAD2.4*	Oeu058547	scaffold782	381	44,185.95	8.62
		*OeuFAD2.5*	Oeu061755	scaffold885	380	43,685.55	8.45

**Table 2:** The numbers of FAD genes in different diploid plants ^1^.

Groups	*A. thaliana*	*O. sativa*	*G. max*	*B. rapa*	*B. oleracea*	*J. regia*	*O. europaea* cv. Farga	*O. europaea* var. Sylvestris
FAB2/SAD	7	8	5	7	6	9	10	7
Total soluble FAD	7	8	5	7	6	9	10	7
ADS	9	0	2	20	18	1	4	1
DES	1	1	2	1	1	2	1	2
FAD4	1	1	0	4	4	1	3	2
SLD	2	1	6	4	5	3	7	5
ω-3	3	4	9	6	7	8	7	5
ω-6	2	4	17	3	3	6	8	5
Total membrane-bound FAD	18	11	36	38	38	21	30	20
Total FAD	25	19	41	45	44	30	40	27

^1^ The FAD genes in *A. thaliana*, *O. sativa*, *G. max*, *B. rapa*, *B. oleracea* and *J. regia* were obtained from The Arabidopsis Information Resource [12], Chi et al. [13], Xue et al. [14] and Liu et al. [16], respectively.

## Data Availability

Not applicable.
